# Investigations for the Possible Use of a Monoclonal Antibody Produced against *Strongyloides ratti* Antigen as an Immunodiagnostic Reagent for Active Strongyloidiasis

**Published:** 2018

**Authors:** Aliyu MAHMUDA, Faruku BANDE, Noor ABDULHALEEM, Roslaini ABD MAJID, Rukman AWANG HAMAT, Wan OMAR ABDULLAH, Zasmy UNYAH

**Affiliations:** 1. Dept. of Medical Microbiology and Parasitology, Faculty of Medicine and Health Sciences, Universiti Putra Malaysia, 43400 UPM, Serdang, Malaysia; 2. Dept. of Parasitology and Entomology, Faculty of Veterinary Medicine, Usmanu Danfodiyo University, Sokoto, Nigeria; 3. Dept. of Pathology and Microbiology, Faculty of Veterinary Medicine, Universiti Putra Malaysia, 43400 UPM, Serdang, Malaysia; 4. Dept. of Biology, College of Science, University of Anbar, Anbar, Iraq; 5. Dept. of Medical Sciences, Faculty of Medicine and Health Sciences, Islamic Science University of Malaysia, Kuala Lumpur, Malaysia

**Keywords:** Monoclonal antibody, *Strongyloides ratti*, Antigen, Active strongyloidiasis, Visceral toxocariasis, Immunodiagnostic reagent

## Abstract

**Background:**

Currently, most of the available serological diagnostic kits for strongyloidiasis are based on the use of the crude antigens of *Strongyloides ratti*, which are good, but with less sensitivity towards the infection. Hence, this study aimed to produce and evaluate monoclonal antibody for detecting soluble parasite antigen in animal sera.

**Methods:**

The study was conducted in the Department of Medical Microbiology and Parasitology, University Putra Malaysia in 2014–2017. Saline extract protein from the infective larvae of *S. ratti* was used to immunize BALB/c mice and subsequent fusion of the B-cells with myeloma cells (SP2/0) using 50% PEG. The hybridomas were cultured in HAT medium and cloned by limiting dilutions. Positive hybrids were screened by indirect ELISA. The ascites fluid from the antibody-secreting hybridoma was purified and the MAb was characterized by western-blots and evaluated in sandwich ELISA for reactivity against the homologous and heterologous antigens.

**Results:**

An IgG1 that recognizes a 30 and 34 kDa protein bands was obtained. The MAb was recognized by all *S. ratti-*related antigens and cross-reacted with only *Toxocara canis* antigens in both assays. The minimum antigen detection limit was found to be 5 ng/ml. All antibody-positive rat and dog sera evaluated have shown antigen-positive reactions in Sandwich-ELISA.

**Conclusion:**

The MAb produced, was able to detect antigens in strongyloidiasis and toxocariasis in animal models and may also be useful for the serological detection of active strongyloidiasis and visceral toxocariasis in human sera.

## Introduction

Current methods for diagnosis of strongyloidiasis are still unsatisfactory due to a wide range of shortcomings ranging from lack of visible clinical signs in patients (1) and irregular worm-egg output during fecal examination (2). There are also practical limitations of the available serological techniques which are only based on antibody detection using crude extract in ELISA which fail to detect active infection (3).

Clinical diagnosis of strongyloidiasis using ELISA test kits containing extract from *S. ratti* is also available, but mainly for antibody detection assays. The use of a crude extract from the filariform larvae of *S. ratti* as antigen in ELISA have always yielded a false positive result (cross-reactivity) in sera of patients infected with other helminth parasites (4, 5). Molecular techniques (PCR) are known to be sensitive and specific, but have been reported to be very expensive and time-consuming (6). As such, alternative methods have been sought for accurate and rapid diagnosis of active strongyloidiasis.

Our approach for use of monoclonal antibodies (MAbs) was due to several reports on their usefulness as reagents employed in biomedical research, diagnosis of diseases, and in the treatment of diseases like infections and cancer (7). They are also said to be mono-specific antibodies produced by identical immune cells, which are clones of a unique parent cell (8), and have been widely applied in the field of clinical medicine, as they can recognize and bind specifically and strongly with respective antigens (9).

MAb production was reported against the excretory/secretory (ES) antigen of *S. stercoralis* (10) and, although, with shortcomings, it is useful in immunodiagnostics of strongyloidiasis. Similarly, MAb production was also reported against the excretory-secretory (ES) antigen of second-stage larvae of *T. canis* (11) and *T. cati* (12) where hybridoma clones were produced. The results were suggested for application in immunodiagnostics of human toxocariasis and also indicated that the antigen detection system (MAb) provides a useful method for the diagnosis of visceral and ocular larva migrans.

The main objective of this study was to produce and evaluate monoclonal antibody-based enzyme immunoassay for detecting soluble parasite antigen in sera for the diagnosis of active *Strongyloides* infections.

## Materials and Methods

### Parasites and experimental animals

The study was conducted in the Department of Medical Microbiology and Parasitology, University Putra Malaysia, Serdang, Malaysia from 2014 to 2017. Female Sprague Dawley rats of 6 wk of age were used for the establishment of *S. ratti* infection model. Propagation of the infective larvae was conducted by modified fecal filtration technique according to the method (13). Female BALB/c mice of eight weeks of age were used for the production of monoclonal antibody, preparation of cloning/expansion media and ascites fluid generation. Anti-SE polyclonal antibodies were generated using female rabbits of 8 wk of age.

### Preparation of saline extract protein from infective larvae and other stages

All antigen types (saline extract and excretory/secretory) from the freshly harvested infective larvae (iL3) and other stages (parasitic females (PF) and free-living stages which includes L1 - L4 and free-living (FLs) adult male and females) were separately prepared, based on the procedure (14). The prepared protein (saline extract) solution was supplemented with fresh protease inhibitor mix and the protein concentration was determined by Bradford Assay (15), then stored in small aliquots of 150 μl at −80 °C until required.

### Preparation of heterologous antigens

All heterologous antigen extracts were prepared. Saline extracts and excretory/secretory products from adult *T. canis* (16) respectively. Saline extract from the filariform larvae of *Ancylostoma caninum* (17). Saline extract from adult *Ascaris suum* (18). Tachyzoites of the RH-Strain of *Toxoplasma gondii* from infected monolayers of Vero cells were also harvested by the procedure (19). All of the heterologous antigens were used for the cross-reactivity study against the candidate monoclonal antibody.

### Preparation of anti-ES polyclonal antibodies

The saline extract antigen (0.05 mg) was prepared with Freund’s complete adjuvant (Sigma-Aldrich, USA) and was used for subcutaneous injection of two white female New Zealand rabbits. Booster immunizations at 2 and 3 wk intervals were given to the rabbits using same antigen emulsified in Freund’s incomplete adjuvant (Sigma-Aldrich, USA). After the third week, sufficient rise in anti-SE antibody titer was confirmed by indirect ELISA. Rabbit sera were prepared, aliquoted and stored at −80 °C until use.

### Indirect ELISA

An indirect ELISA was used to screen for antibodies of the hybrid cells in spent media, and for quantitative determination of antibodies in infected rats and puppies and in immunized mice and rabbits. Plates (Corning Incorporated, USA) were coated with 150 μL/well of both antigen extracts (*S. ratti*, *T. canis*) at a concentration of 200 ng/ml in carbonate bicarbonate buffer (pH 9.6) then incubated at 4 °C overnight. The unbound antigen was washed away using a TBS buffer (pH 8.0) containing 0.05% Tween 20. The uncoated sites were blocked with PBS buffer containing 1% Tween 20 and 10% Bovine Serum Albumin and incubated (37 °C, 2 h). After washing, 100 μL of the positive sera of infected rats and puppies, immunized mice sera, immunized rabbit (1:1000) or individual spent media from wells containing clones were added to appropriate antigen-coated wells. The plates were incubated (37 °C, 1 h) then washed. A volume of 100 μL of 1:10000 dilution of a horseradish peroxidase (HRP) conjugated goat anti-mouse IgG, goat anti-Rat IgG, goat anti-Canine IgG secondary antibody and goat anti-rabbit IgG (Thermo Fisher Scientific, USA) in buffer was added to all wells and the plates were further incubated (37 °C, 1 h). After the final wash, 100 μL of 1 X TMB peroxidase substrate (Thermoscientific, USA) was added to all wells. The enzymatic reaction was allowed to take place at room temperature in the dark for 30 min. The reaction was stopped by adding 100 μL/well of 0.12 M HCl and the optical density (OD) was measured at the absorbance of 450 nm using a microplate reader.

### Preparation of monoclonal antibody

Monoclonal antibodies were prepared by the method (20). Eight weeks-old Balb/C mice were given an initial priming dose of 150 μL (100 μg) of the saline extract antigen emulsified in complete Freund’s adjuvant (CFA), using a sterile syringe by intraperitoneal injection. The first immunization was followed by two booster doses with the same antigen emulsified in incomplete Freund’s adjuvant (IFA) after two and three weeks respectively. Five days after the last immunization, the antibody titer of the immunized mice was confirmed by indirect ELISA. The mouse with the highest antibody titer was selected as a source of spleen cells for fusion with myeloma cells while the other immunized mice were bled and their sera pooled for use as positive control sera. A booster dose with only protein in 1 x PBS was given intraperitoneally three times, three days in a row and cell fusion was then performed the next day following sacrifice of the selected mouse. Spleen cells were isolated and fused with myeloma (SP2/0) cells at a ratio of 10 spleen cells to 5 myeloma cells. Subsequently, hybridomas were selected by culturing the fused cells in media supplemented with 10% Hypoxanthine Aminopterin Thymidine (Sigma Aldrich, USA). Hybrid cells were screened for positivity by Indirect ELISA and hybridomas secreting specific antibodies were expanded and cloned by limiting dilution technique. The produced monoclonal antibody was isotyped using a mouse monoclonal isotyping (MMT1) kit (BioRad, USA). Finally, 1 × 10^6^ antibody-producing hybridoma cells were given to eight-weeks-old BALB/c mice for ascites fluid production. The ascetic fluid obtained was purified using a monoclonal antibody IgG purification kit (Thermo scientific, USA).

### Monoclonal antibody characterization using western-immunoblotting

The saline extract that had been resolved in the polyacrylamide gel by electrophoresis was electroblotted onto a sheet of nitrocellulose membrane (NCM) using Trans-Blot^®^ S-D Semi-Dry Electrophoretic Transfer Cell (Bio-Rad, USA), according to the Manufacturer’s instructions. After blotting, the empty sites on the NCM were blocked by soaking the membrane in the blocking solution (25 ml, pH 7.4) of 0.01% Antifoam A and non-fat dry milk in TBS buffer (Thermo Scientific, USA) at room temperature with gentle rocking for 25 min. The blocking solution was poured off and NCM was then washed twice with 50 ml of TBS-Tween-20 wash buffer (Thermo Scientific, USA). It was then placed in a solution of 1:1000 of the monoclonal antibody preparations in blocking buffer for 20 min at room temperature on a rocking platform. Thereafter, the NCM was washed twice with 50 ml of TBS-Tween-20 wash buffer (Thermo Scientific, USA) as above and then transferred into 10 ml of a solution of anti-mouse immunoglobulin-horseradish peroxidase conjugate (Thermo Scientific, USA) at 1:10000 in blocking buffer for 15 min at room temperature with continuous shaking. After washings twice to remove excess antibody, the NCM was placed in 10 ml of a freshly prepared substrate (TMB blotting solution) for 30 min until bands of immune complexes appear. The NCM was then washed twice with distilled water until the background is visibly clear, and the paper was air dried for 30 min.

### Monoclonal antibody-based Sandwich-ELISA for detecting antigens in sera of infected animals

Wells of microtiter plates were coated with a 1: 1000 dilution of monoclonal antibody (10 ng/ml) supernatant in bicarbonate buffer and were incubated at 4 °C overnight. Plates were washed three times with wash buffer (PBS-T) and were blocked with 300 μL/well of blocking buffer and incubated (37 °C, 1 h). After washing three times, different concentrations of the saline extract antigen of 0, 0.5, 1, 5, 10, 50, 100 and 150 ng/ml with the blocking buffer were made. These dilutions and a 1:1000 dilution of 20 non-infected rats’ sera (negative control) were used for capture ELISA. Same concentrations (5 ng/ml) of heterologous antigens (*T. canis*, *A. caninum*, *A. suum* and *T. gondii*) along with 3 other antigens of *S. ratti* and 1:1000 dilution of sera from infected rats and dogs in blocking buffer were added to the wells (100 μL/well), incubated (37°C, 1 hr) and washed with wash (PBS-T) buffer. The anti-SE rabbit polyclonal antibody was used as the second antibody (1:1,000) following incubation at 37 °C for 1 h. A volume of 100 μL of 1:1000 dilution of a horseradish peroxidase (HRP) conjugated goat anti-rabbit IgG antibody (Abcam, UK) in buffer was added to all wells and the plates were further incubated at room temperature for 10 min. After the final wash, 100 μL of 1 X TMB peroxidase substrate (Thermo scientific, USA) was added to all wells and incubated at room temperature in the dark for 30 min. The reaction was stopped by adding 100 μL/well of 0.12 M HCl and the optical density (OD) was measured at the absorbance of 450 nm using a microplate reader. The results were declared positive when the read-out optical density (OD) is greater than mean + 3 standard deviations of absorbance values of the negative control sera.

## Results

**Fig. 1:**
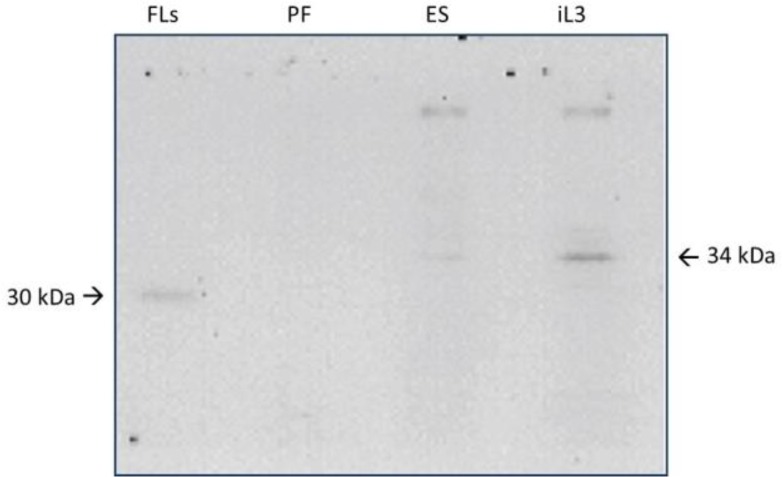
Western Immunoblotting of MAb-P38-C5 against antigens of *S. ratti*: FLs = free-living stages (saline extract); PF = Parasitic females (saline extract); ES = Excretory/secretory product from iL3; iL3 = Saline extract from iL3

**Fig. 2:**
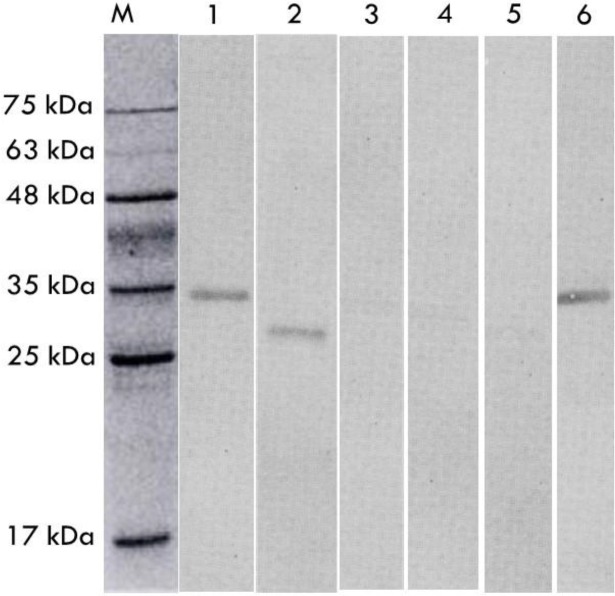
Cross reactivity study of MAb-P38-C5 against other parasite antigens. M = Protein ladder; 1 = *T. canis* (SE); 2 = *T. canis* (E/S); 3 = *A. caninum*; 4 = *A. suum*; 5 = *T. gondii*; 6 = *S. ratti*

A monoclonal antibody against the saline extract of *S. ratti* was obtained (MAb-P38-C5). The isotype of the produced monoclonal antibody was IgG1. It was checked against all *S. ratti*-related antigens (saline extracts from free-living stages, parasitic females and ES from infective larvae) as well as antigens from other parasites (*T. canis*, *A. caninum*, *A. suum* and *T. gondii*) to determine cross-reactivity. Western blot results of the monoclonal antibody (MAb-P38-C5) revealed bands of immune complex against the SDS-PAGE separated antigens of *S. ratti*. Interestingly, reactive to 34 kDa protein band of both saline extract and ES product from the infective larva (iL3) and against 30 kDa protein band of the saline extract from the free-living (FL) stages, but did not reveal any reactivity against the saline extract from the parasitic (PF) females (Fig. 1). To determine further specificity, MAb-P38-C5 did not react with the antigens of *Ancylostoma*, *Ascaris*, and *Toxoplasma,* but reacted with a 30 kDa protein of *T. canis* ES antigen and 34 kDa protein of the *T. canis* saline extract antigen in western blots (Fig. 2).

While using negative sera from twenty (20) non-infected rats in sandwich ELISA, the result indicated a mean absorbance value (OD) of 0.135 obtained at 450 nm with a standard deviation (SD) of 0.039. A cut-off value of 0.252 was established as Mean+3 Standard deviations. Accordingly, OD values greater than 0.252 were considered as positive while those less than 0.252 as negative. Using the criteria above, sandwich ELISA experiments using *S. ratti* saline extract (SE) antigen with eight different antigen concentrations which include 0 mg/ml, 0.5 ng/ml, 1 ng/ml, 5 ng/ml, 10 ng/ml, 50 ng/ml, 100 ng/ml and 150 ng/ml was performed.

**Fig. 3:**
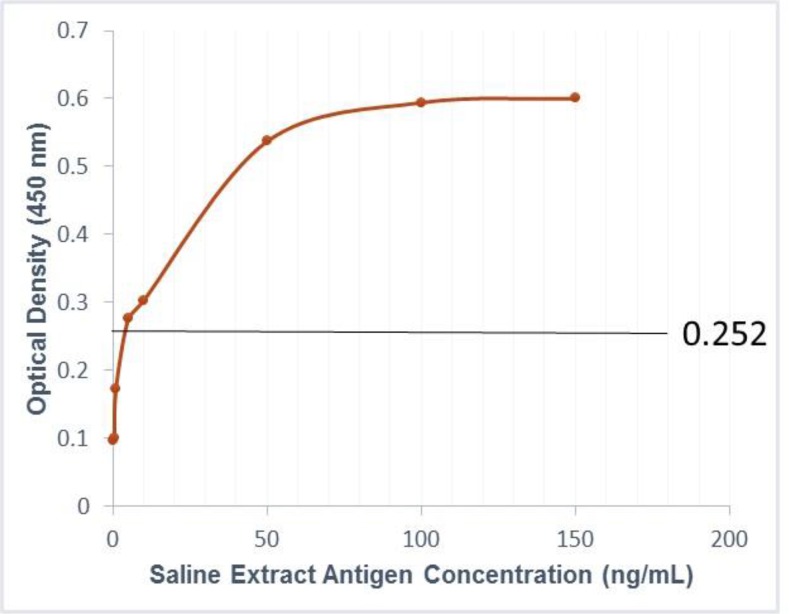
Relationship between *S. ratti* antigen (saline extract) concentration and absorbance values showing a cut-off value (Mean absorbance value + 3 standard deviations) from 20 negative rat sera

This is to determine the minimum antigen detection limit of the candidate MAb. Results yielded OD values showing a nearly linear distribution which is dependent on the antigen concentration. Hence, because the OD value obtained at 5 ng/ml of the saline extract (SE) was 0.276, just little above the cut-off value, the minimum antigen detection limit of this method was considered to be 5 mg/ml (Fig. 3).

**Fig. 4:**
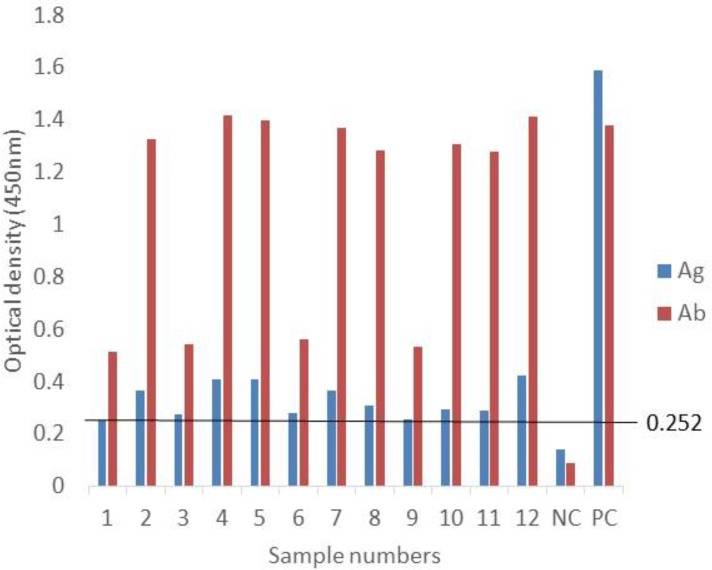
Antigen levels in 12 *S. ratti* antibody positive rat sera. Ag= antigen; Ab= antibody; NC= negative control serum; PC= positive control serum

**Fig. 5:**
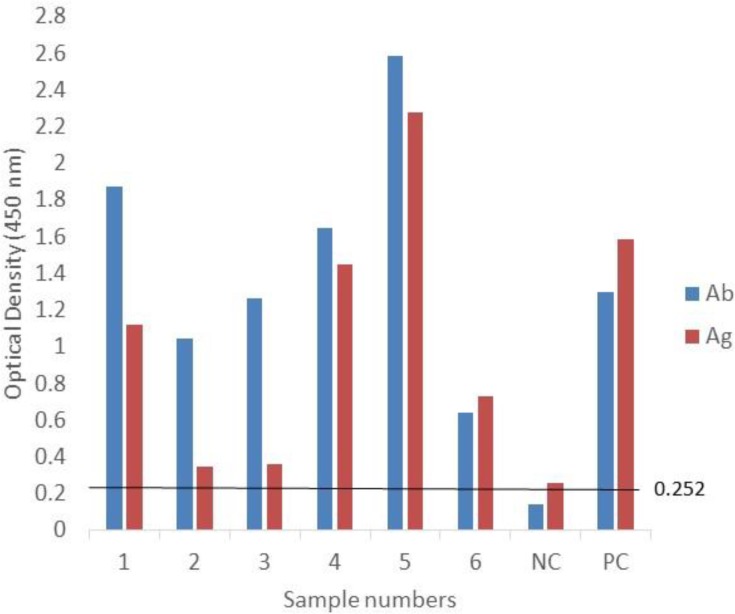
Antigen levels in 6 *T. canis* antibody positive dog sera. Ag= antigen; Ab= antibody; NC= negative control serum; PC= positive control serum; Cut-off value = 0.252

Subsequently, the MAb-P38-C5 was used in Sandwich-ELISA for detection of *S. ratti* antigen in sera. The monoclonal antibody reacted with all *S. ratti*-related antigens and *T. canis* antigens in capture ELISA but did not react with the antigens of the other parasites (Table 1). Interestingly, all the twelve indirect ELISA *Strongyloides* positive rat sera from infected rats examined have shown antigen-positive reactions in sandwich ELISA (Fig. 4). Similarly, all six indirect ELISA Toxocara positive dog sera were also antigen-positive in sandwich ELISA (Fig. 5).

**Table 1:** Cross-reactivity of MAb-P38-C5 against heterologous antigens by Sandwich ELISA

***S/No***	***Antigens***	***OD Values***
1	*Strongyloides ratti* (ES) iL3	0.573*
2	*Strongyloides ratti* free-living stages	0.349*
3	*Strongyloides ratti* parasitic females	0.314*
4	*Toxocara canis* (SE)	0.298*
5	*Toxocara canis* (E/S)	0.277*
6	*Ancylostoma caninum*	0.219
7	*Ascaris suum*	0.164
8	*Toxoplasma gondii*	0.079

Cut-off value = 0.252;

* = positive

## Discussion

In this study, an attempt to produce monoclonal antibody specific to the saline extract antigen of *S. ratti* was made potentially used in immunodiagnosis of active strongyloidiasis. The monoclonal antibody produced designated MAb-P38-C5 reacted with the antigens of *S. ratti* and *T. canis* in both western-immunoblots and capture ELISA, which indicates that it could be used for antigen detection assay in strongyloidiasis and visceral toxocariasis. The processed larvae (saline extract) antigen contains multiple, well-defined specific molecular weights of *S. ratti* protein bands. The antibody was reactive to 30 kDa and 34 kDa protein bands in western-immunoblots analysis against the SDS-PAGE separated homologous antigen.

Furthermore, the hybrid produced, secreted kappa IgG1 immunoglobulin which is the iso-type known to have high binding affinity to their epitopes. IgG1 was said to be among the major components of the immunoglobulin response in strongyloidiasis (21) and was also seen to be up-regulated in early infections, but declined as the infection becomes chronic (22). High levels of specific IgG1 were higher in patients with strongyloidiasis than in patients infected by other helminths and its recognition is more exact compared to detection of total IgG in patients with strongyloidiasis (23). This was suggested to be because of its diagnostic significance in immunological tests and protective role for *Strongyloides* infections (24). Moreover, significantly, human infection with *S. stercoralis* has been reported to result in a specific antibody response, mainly of the IgG class (6).

Detection of only one band indicates that a single epitope of the antigen was recognized by the antibody while detection of more than one band indicates that several epitopes might have been recognized. This finding is still valuable for diagnostic purpose, provided that these two epitopes are specific sites for the parasite to be detected. One band using western blot was detected (20), 2–4 bands were detected from a MAb in SDS-Polyacrylamide gel electrophoresis (25) and reactivity of MAb to three different bands was reported in western blot (11), some of which were free from cross-reactivity with parasites other than the homologous antigen.

Interestingly, the reactivity of the MAb to these protein antigens in western-immunoblots has been previously reported. These antigenic components include protein of 28–35 kDa size (26). In addition, among the thirteen immunodominant bands of the *S. ratti* soluble extract recognized by serum IgE which include a 28 and 36 kDa (27) and a 36 kDa band (28) may also correspond to 30 and 34 kDa bands described in this study. Soluble protein of 25, 30 and 40 kDa were recognized by IgG in sera of strongyloidiasis patients on western-immunoblots of soluble larval extracts of *S. stercoralis* (22) which also supports the studies demonstrating the reactivity of serum IgG against 28, 31 and 41 kDa protein bands (29) and protein of 26 and 41 kDa (4). The 30 and 31 kDa protein may correspond to the 30 kDa protein found in this current study.

The observation of cross-reactivity in western-immunoblots of the MAb-P38-C5 with *T. canis* antigens at 34 kDa were, however, not strange as similar reports of serum IgG reactivity against both 35.70 kDa and 33.77 kDa bands from adult *T. canis* somatic protein and excretory/secretory protein antigens respectively were also documented (16). Similarly, a similar serum IgG reactivity was reported in western-immunoblots against 30 and 35 kDa protein of *T. canis* excretory/secretory (ES) protein (30), 24, 28, 30 and 35 kDa bands of *T. canis* ES antigen (31) while a reactivity of an IgG1-MAb specific against a 30 kDa band of *T. canis* excretory/secretory protein was observed (11). This similarity may be due to the conformational configuration on the epitopic sites of the respective antigen molecules.

The present results showed that the capture ELISA was however found to be sensitive enough to detect as little as 5 ng/ml of *S. ratti* antigen (SE), as a minimum detection limit. This provides sufficient sensitivity for the diagnosis of strongyloidiasis. It is lower than the level of 78 ng obtained using dot-ELISA against the E/S antigen of *S. stercoralis* (10) but similar to the minimum concentration of 5 ng/mL obtained for both *T. canis* (11) and *T. cati* (12). Saline extract from the free-living stages of *S. ratti* with reactivity in western-immunoblots also had a positive absorbance reading in capture ELISA. Antigen of the parasitic females of *S. ratti* although negative in the western-immunoblots, reacted with the antibody (MAb) in capture ELISA. This is similar to the report (10) where a monoclonal antibody against the excretory/secretory protein of *S. stercoralis* was found reactive to the antigen in Dot-ELISA but non-reactive in western blot. The reason for this variation may be due to the structural integrity of the proteins in the assays. In western immunoblotting, a higher concentration of the antigen is used (30 μg/well) compared to that in capture ELISA (5 ng/ml).

Furthermore, the proteins are heated to 80 °C for 15 min and denatured by the SDS in order to pass effectively through the acrylamide gel while in ELISA they remain in their natural conformation bound to the plates. This difference, therefore, might create two unique epitopes to recognize, resulting in the antibody being non-reactive to the denatured version in western-immunoblots but reactive to the stable version in Sandwich-ELISA. These similarities and differences in the reactivity of the MAb against the antigens in both assays further highlight the significance of the characterization process.

The comparison between antibody and antigen detection in twelve immunosuppressed rats infected with *S. ratti* and six puppies infected with *T. canis* have shown that these animals were having active *Strongyloides* and *Toxocara* infections, respectively. For circulating antigen detection, a wide variety of combinations of antibody, conjugates, and dilution rates have been documented (20, 31, 32). Use of MAbs for sensitization has been recommended because cross-reaction is likely to occur due to similar molecular weights protein bands shared among related helminth parasites (25). Studies have also reported evaluation of a Sandwich-ELISA method with a decrease or no cross-reactivity and with high diagnostic efficiency (8, 33).

Our findings using Sandwich-ELISA with the MAb indicated that the test only cross-reacted with *Toxocara* and can also detect as little as 5 ng/ml of *Strongyloides* and *Toxocara* antigens. This enables a highly reliable detection of circulating antigens in sera. Results obtained with experimentally infected immuno-suppressed rats and naturally infected puppies indicated that even very small amounts of antigens can be detected in infected animals. Hence, our Sandwich-ELISA system might fulfill the requirement that any given diagnostic assay must be quite sensitive to pick up a very low level of antigen in sera.

## Conclusion

This study provided the first information on the possible use of saline extract antigen for production and evaluation of monoclonal antibody in an enzyme-immunoassay against *Strongyloides ratti* in an animal model. The cross-reactivity against *Toxocara* antigens is however observed as a limitation which needs to be addressed in future studies possibly through the use of a novel recombinant antigen or short-peptide based proteins that would be able to differentiate between the two infections in the field scenario. Although the candidate MAb is not exclusively specific for *Strongyloides* antigens, it could detect antigens in sera.

## Ethical approval

Use of animals in this experiment was approved by and conducted in accordance with the guidelines of the Animal Care and Use Committee (ACUC), Faculty of Medicine and Health Sciences, University Putra Malaysia, Serdang Malaysia (UPM/FPSK/PADS/BRUUH/00247).
